# The Impact of Exercise on Interleukin-6 to Counteract Immunosenescence: Methodological Quality and Overview of Systematic Reviews

**DOI:** 10.3390/healthcare12100954

**Published:** 2024-05-07

**Authors:** Anne Sulivan Lopes da Silva Reis, Guilherme Eustáquio Furtado, Marcos Rodrigo Trindade Pinheiro Menuchi, Grasiely Faccin Borges

**Affiliations:** 1 Postgraduate Program in Physical Education, The State University of Santa Cruz (PPGEF/UESB/UESC), Ilhéus 45650-000, BA, Brazil; diploanne@gmail.com (A.S.L.d.S.R.); mrtpmenuchi@uesc.br (M.R.T.P.M.); 2 Polytechnic Institute of Coimbra, Applied Research Institute, Rua da Misericórdia, Lagar dos Cortiços-S. Martinho do Bispo, 3045-093 Coimbra, Portugal; guilhermefurtado@ipg.pt; 3 Research Centre for Natural Resources Environment and Society (CERNAS), Polytechnic Institute of Coimbra, Bencanta, 3045-601 Coimbra, Portugal; 4 Sport Physical Activity and Health Research & Inovation Center, 4960-320 Melgaço, Portugal; 5 Center for Public Policies and Social Technologies, Federal University of Southern Bahia, Praça José Bastos, s/n, Centro, Itabuna 45600-923, BA, Brazil

**Keywords:** systematic reviews, immunosenescence, exercise, interleukin-6 receptors, immune system

## Abstract

Objective: This study evaluated the methodological quality of published systematic reviews on randomized and non-randomized clinical trials to synthesize evidence on the association between IL-6, immunosenescence, and aerobic and/or resistance exercise. Method: The Preferred Reporting Items for Overviews of Systematic Reviews (PRIO-harms) guideline was used, with registration number CRD42022346142-PROSPERO. Relevant databases such as Cochrane Library, PubMed, Web of Science, Scopus, and Google Scholar were searched using English Medical Subject Headings terms. Inclusion criteria were systematic reviews analyzing aerobic exercise, resistance exercise, or a combination of both and assessing IL-6 as a biomarker of cellular immunosenescence in humans. The Measurement Tool to Assess Systematic Reviews 2 (AMSTAR-2) was employed. Results: Out of 742 identified articles, 18 were eligible, and 13 were selected for analysis. Sample sizes ranged from 249 to 1421 participants, mostly female, with ages ranging from 17 to 95 years. Aerobic exercise was the most studied type (46.15%), followed by combined exercise (38.46%) and resistance exercise (15.38%). Aerobic exercise showed a statistically significant reduction in IL-6, C-reactive protein (CRP), and tumor necrosis factor-alpha (TNF-α) levels. Among the 13 reviews analyzed using AMSTAR-2, 8 were rated as critically low quality, and 5 were classified as low quality. Conclusion: Aerobic exercise has anti-inflammatory properties and the potential to modulate IL-6, CRP, and TNF-α levels in immunosenescence. However, the limited methodological quality of the analyzed systematic reviews highlights the urgent need for robust, high-quality studies to improve access to information and facilitate evidence-based decision-making in healthcare.

## 1. Introduction

Aging, from an evolutionary perspective, can be seen as the cumulative impact of mild and temporarily limited inflammatory adaptive responses to fundamental activities such as nutrition/feeding and regular exercise [1,2,3,4], which, in the face of successful organic adaptations, can result in a general improvement or adequate maintenance of the state of health [5,6,7,8]. On the other hand, changes in immunosenescence, metabolic responses, stress, and the imbalance caused by inflammaging [9,10,11] play significant roles in most chronic diseases associated with aging.

Inflammaging is influenced by single nucleotide polymorphisms (SNPs) in the promoter regions of genes encoding interleukin-6 (IL-6) and interferon-gamma (IFN-γ). Genetic variations in the IL-6 promoter, specifically at the -174C/G locus, and their impact on serum IL-6 levels in older individuals, including centenarians, contribute to inflammaging [10,11,12,13,14,15]. Consequently, the coexistence of elevated autoimmunity and inflammation with immunodeficiency reflects the enigmatic paradox involved in the aging process [16,17]. However, from an evolutionary standpoint, changes in the immune system can be considered a form of adaptation or restructuring rather than solely damaging since an optimized inflammatory response in conjunction with a competent anti-inflammatory network is crucial for successful aging and targeted longevity [10,18,19].

The effects of regular exercise on age-related processes have been confirmed by the scientific field, with a prominent emphasis on the reduction in inflammatory levels, the regulation of immune mechanisms, and the possibility of delaying the onset of cellular senescence [20,21,22,23,24,25,26]. The systemic impact on organs and tissues through the secretion of substances resulting from exercise (exerkines) results in profound therapeutic actions in the immune, cardiovascular, metabolic, and neurological systems [27].

The scientific field has confirmed the effects of regular exercise on age-related processes, particularly in reducing inflammatory levels, regulating immune mechanisms, and potentially delaying cellular senescence [20,21,22,23,24,25,26]. Exercise exerts systemic impacts on organs and tissues through the release of substances known as myokines/exercises [27], such as IL-6, which elicit various beneficial physiological and metabolic changes in skeletal muscles, white adipose tissue, liver, bones, immune cells, and the central nervous system [28,29]. Consequently, exercise can reduce inflammation, enhance cellular insulin sensitivity, regulate energy expenditure, and exert anti-inflammatory actions capable of modulating immune dysfunctions, tumorigenic triggers, and other complexities [23,30,31,32,33,34,35].

It is important to note that IL-6 exhibits both anti-inflammatory and pro-inflammatory actions, along with several hematological, endocrine, and metabolic implications [25,36,37,38]. This multifaceted action accurately portrays its complex signal transduction, characterized by a wide range of effects based on the activation of the receptor system and the constancy of stimulation [29,39]. IL-6 also serves as a crucial mediator between the acute and chronic phases of inflammatory action, influencing specific cellular and humoral immune responses, such as end-stage B-cell differentiation, immunoglobulin secretion, and T-cell activation [25,36]. Moreover, the interleukin-6 receptor (IL-6R) can convert the neutrophilic leukocyte infiltrate (predominant in the acute phase of inflammation) into an abundant lymphocyte infiltrate (characteristic of the chronic phase of inflammation), resulting in elevated IL-6 levels in numerous inflammatory diseases [25,40,41,42].

Although there are a significant number of published systematic reviews examining IL-6, exercise, and immunosenescence [43,44,45,46,47,48,49,50,51,52,53,54,55,56,57,58,59,60,61], these have focused on specific population subgroups, conditions, and particular forms of exercise (e.g., only acute interventions or without variations in intensity). Studies demonstrate diverse effects of exercise on IL-6 levels. Kim and Yeun [44] showed IL-6 reduction in older adults with chronic conditions during resistance exercises, while Zheng et al. [52] observed IL-6 decrease in healthy adults with aerobic exercises. Yang et al. [43] reported reductions in IL-6 levels across populations with combined exercises.

In this sense, the proposal to carry out the most comprehensive synthesis to date, with evidence on the effects of all types of physical exercise on IL-6 in the processes of immunosenescence, is very pertinent. Accordingly, in the absence of an overview summarizing these reviews, this study aims to systematically gather, evaluate, and synthesize scientific evidence in order to improve access to information and support health decision-making [62,63,64]. Therefore, this study focuses on evaluating the methodological quality of published systematic reviews that include randomized and non-randomized clinical trials to synthesize evidence on IL-6, immunosenescence, and aerobic and/or resistance exercise.

## 2. Materials and Methods

### 2.1. Overview Conduct

This is an overview of systematic reviews conducted in accordance with the guidelines outlined in the Cochrane Handbook for Systematic Reviews of Interventions [65]. The reporting of this overview follows the Preferred Reporting Items for Overviews of Systematic Reviews (PRIO-harms) [66] and the Preferred Reporting Items for Systematic Reviews and Meta-Analyses (PRISMA) [67]. The study protocol for this overview has been registered in PROSPERO—the International Prospective Register of Systematic Reviews with the registration number CRD42022346142.

### 2.2. Eligibility Criteria

For this overview, original articles from systematic reviews, with or without meta-analysis, that met the eligibility criteria were considered. Only systematic reviews that included randomized clinical trials or non-randomized clinical trials, published in the English language, and focused on aerobic and/or resistance exercise or a combination of both, with IL-6 as a biomarker of cellular immunosenescence in humans, were included for analysis. The participant population encompassed several age groups, including adolescents (17 years), adults (19–64 years), and the elderly (>65 years), irrespective of gender. Studies that reported inconsistent methodology, the absence of research/intervention procedures, or were directly related to coronavirus (COVID-19/SARS-CoV-2) were excluded.

### 2.3. PICO Framework Application

It is important to note that the inclusion criteria were based on the PICO framework, addressing the following question: Do people who engage in aerobic and/or resistance exercise demonstrate differences or improvements in IL-6 levels during the process of immunosenescence compared to those who do not exercise? The elements of the PICO are set out in Table 1.

### 2.4. Data Selection and Extraction

The studies were independently selected by two authors (A.S.L.d.S.R. and A.C.S.S.) by reading the titles and abstracts in the index databases Cochrane Library, PubMed, Web of Science, Scopus, and the Google Scholar search tool in July 2022, with updates in February 2023. There were no restrictions regarding the publication date. High-sensitivity strategies were employed in the search, utilizing Medical Subject Headings (MeSH) terms in English along with keywords in the search strategy.

### 2.5. Search Strategy

The search strategy was adjusted for each database or portal accessed. Boolean operators “AND” and “OR” were used, along with the specific search strategy for systematic reviews in PubMed, with minor adjustments. Additionally, the references to the included studies were analyzed to expand the retrieval of relevant data. The descriptors “Exercise”, “Cellular Senescence”, “Cytokines”, “Inflammation”, “Systematic Review”, and “Aging”, along with their variations, were utilized to enhance the sensitivity of the search strategy, as shown in Table 2.

### 2.6. Data Extraction

Data extraction was independently conducted by three authors (A.S.L.d.S.R., A.C.S.S., and G.G.C.L.), who thoroughly reviewed the included studies. Any discrepancies were resolved through consultation with a fourth experienced author (G.F.B.). The Rayyan QCRI online platform was utilized for data selection and extraction [68]. Mendeley reference management v.1.19.8 software facilitated storage, organization, sharing of records, and removal of duplicates. Data were meticulously organized in a Microsoft Excel^®^ 2019 Plus spreadsheet, incorporating various variables, including portal, author, year, country of the sample, end date of collection, PICO, registration protocol, sample, gender, age, type of intervention, duration, control/intervention groups, extraction method, health condition, biological samples, immunological markers, software, tests, primary and secondary outcomes, evaluation instrument/methodological quality, and conclusion.

### 2.7. Methodological Quality Assessment

The methodological quality assessment of systematic reviews was conducted by two authors (A.S.L.d.S.R and G.F.B.) using the Measurement Tool to Assess Systematic Reviews 2 (AMSTAR-2). This critical appraisal tool for systematic reviews of randomized or non-randomized studies consists of 16 items, with seven (I.2, I.4, I.7, I.9, I.11, I.13, I.15) considered critical domains, and eight (I.1, I.3, I.5, I.6, I.8, I.10, I.12, I.16) considered non-critical. The quality rating is classified as follows: high (no or a single non-critical weakness), moderate (more than one non-critical weakness), low (one critical failure with or without non-critical weaknesses), and critically low (more than one critical failure with or without non-critical weaknesses) [69].

## 3. Results

**Figure 1:**
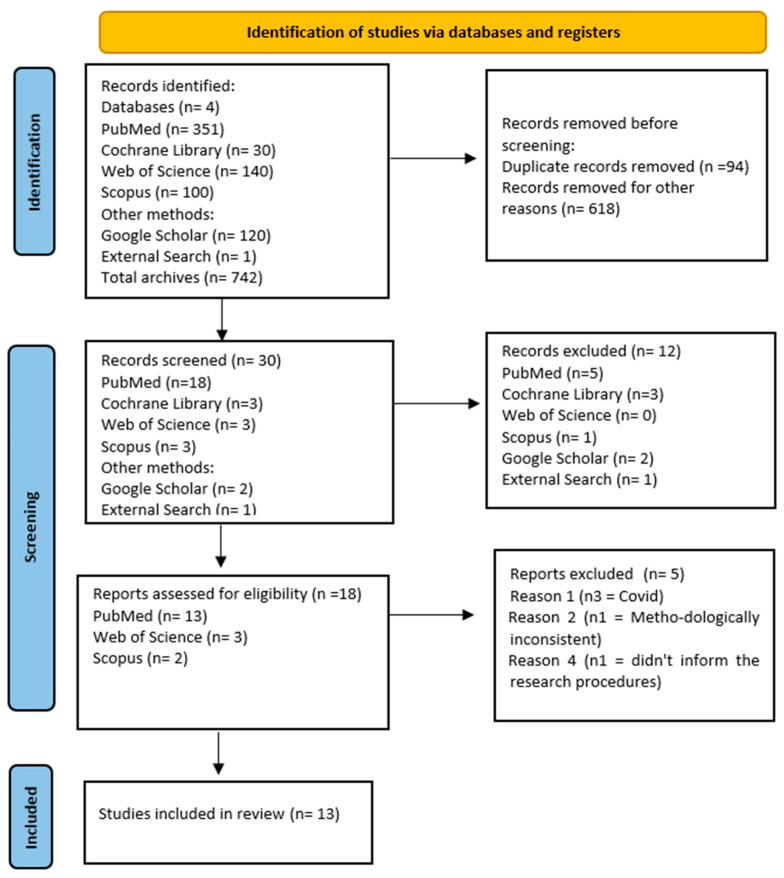
Flowchart of the study selection process.

Initially, a total of 742 scientific articles were identified in the Cochrane Library, PubMed, Web of Science, Scopus, and Google Scholar. Of these, 618 files were removed before screening due to duplicates. During the screening process, 18 files met the eligibility criteria, but five articles were subsequently excluded. Among the excluded articles, three were related to coronavirus (COVID-19/SARS-CoV-2), one exhibited methodological inconsistency, and one lacked research/intervention procedures. Following a thorough reading of the articles, a total of 13 systematic reviews were included in the final analysis. The entire selection process, along with the reasons for exclusion, can be found in the flowchart (Figure 1).

Of the 13 systematic reviews included [43,44,45,46,47,48,49,50,51,52,53,54,55,56,57,58,59,60,61], six presented meta-analyses [43,44,50,52,58,60]. All reviews assessed the methodological quality of the included studies, and the instruments used were Cochrane Collaboration/RoB/RoB2/ROBINS-I (38.46%; n = 5) [43,44,45,46,52], Jadad scale (15.38%; n = 2) [60,61], PEDro scale (15.38%; n = 2) [50,59], NICE guidelines (7.69%; n = 1) [47], Downs and Black Scale (Modified) (7.69%; n = 1) [54], Quality Rating Scale (7.69%; n = 1) [51], and an uninformed instrument (7.69%; n = 1) [58].

The year of publication of the systematic reviews ranged from 2008 to 2023, with eight of them including randomized clinical trials [43,44,50,52,58,59,60,61] and five including both randomized and non-randomized clinical trials [45,46,47,51,54]. The number of studies included in the systematic reviews ranged from 11 to 76. The sample sizes ranged from 249 to 1421 participants, with the majority being female. The age range varied from 17 to 95 years old. The main diseases mentioned were cancer, diabetes, and hypertension. The most commonly performed exercises were aerobics (46.15%; n = 6) [51,52,54,58,59,60] combined with 38.46% (n = 5) [43,46,47,50,61] (aerobic + resistance) and resistance (15.38%; n = 2) [44,45]. The modalities most frequently used were walking, running, cycling, swimming, Tai Chi, yoga, and weight training.

The duration of interventions ranged from 4 days to 104 weeks. The most commonly used methods of analysis in the studies were blood sample analysis (n = 5) [43,50,54,59,61], flow cytometry (n = 3) [45,46,60], and an uninformed method (n = 5) [44,47,48,51,52]. The most prominent endpoints observed were related to inflammatory conditions, specifically IL-6, CRP, and TNF-α (Table 3).

### 3.1. Resistance Exercises

Kim and Yeun [44] demonstrated in their systematic review, which included 18 studies with 539 participants aged 62.0 to 82.7 years old, that moderate-intensity resistance exercise performed two to four times per week for durations of 20 to 70 min over a 12-week period had a significant effect on reducing IL-6, CRP, IL-10, and TNF-α levels in older individuals with chronic conditions such as cancer and diabetes. The majority of participants in the studies were female. Similarly, Salimans et al. [45] conducted a study that included 30 studies with 835 participants aged 17 to 85 years old, with a sample of healthy individuals, primarily women, spanning a wide age range. They found that acute/chronic resistance exercise did not lead to significant changes in IL-6, IL-6R, or TNF-α levels. However, they did observe the activation of the nuclear factor kappa B (NF-κB) signaling pathway in peripheral blood mononuclear cells (PBMC) and an increase in MCP-1 levels.

### 3.2. Aerobic Exercises

The review by Zheng et al. [52], which included 11 studies with 1250 participants aged 40 to 95 years old, demonstrated a significant impact on decreasing levels of IL-6, CRP, and TNF-α, but with aerobic exercise, equally moderate intensity, with two to five sessions per week, and a duration of 20 to 90 min. The sample was analyzed with healthy people, the majority being female. In contrast, the study by Barros et al. [54], which included 51 studies with 1421 participants and an average age of 39.16 years old, shows increased serum levels of IL-10 and TNF-α and decreased amounts of IL-6 in athletes and non-athlete runners, with a majority male gender, in acute and chronic sections of this sport. This, therefore, indicates aerobic exercise as a possible mediator in the processes involving pro-inflammatory and anti-inflammatory markers.

A review conducted by Tong et al. [58], which included 76 studies with 276 participants aged 41.5 to 70.43 years old, also found that aerobic exercises in a population diagnosed with cancer, although admitting slight changes in CD4+, CD4+/CD8+, CD3+, NKCA, and IL-2, failed to show combined effects on IL-6, CD8+, and TNF-α. In contrast, the systematic review by Djalilova et al. [51], which included 15 studies with 937 participants aged 35 to 66 years old, with a mostly female sample diagnosed with cancer and practicing aerobic exercise, observed a noticeable decrease in inflammatory aspects associated with this chronic condition, but with inexpressive effects for IL-6, CRP, and TNF-α.

The study by Ploeger [59], which included 19 studies with 316 participants aged ≤ 18 years old, observed that chronic aerobic exercise sessions practiced by people with inflammatory diseases, specifically type I diabetes mellitus, cystic fibrosis, and chronic obstructive pulmonary disease, when compared to healthy controls, favored the worsening of such conditions due to the increase in pro-inflammatory markers TNF-α and IL-6 right after the exercise. However, in people with chronic heart failure and type 2 diabetes mellitus, chronic aerobic exercise was shown to attenuate the systemic inflammatory process. In the same direction, Ng and Tsang [60], which included 26 studies with 796 participants, 18.5 to 77.5 years old, suggest that long-term aerobic exercise in a population with various chronic dysfunctions tended to reduce IL-6 levels and increase the number of white blood cells and lymphocytes, with perceptions of providing regulation of the sympathetic and parasympathetic nervous systems.

### 3.3. Combined Exercises

The study by Yang et al. [43], which included 18 studies with 853 participants aged 40 to 55 and >55 years old, demonstrated significant reductions in the levels of IL-6, IL-10, CRP, and TNF-α through interventions of aerobic, resistance, combined, and high-intensity interval exercise (HIIT), with durations ranging from 3 weeks to 12 months. The study included a sample balanced by both sexes in the middle-aged/elderly age group with type 2 diabetes mellitus. However, aerobic and combined exercise showed superiority over resistance exercise and HIIT in decreasing IL-6 and IL-10 levels, while HIIT was superior in reducing TNF-α levels.

Bautmans et al. [47], which included 19 studies with 985 participants aged 56 to 83 years old, also obtained positive results in reducing IL-6, CRP, and TNF-α levels in a population consisting of both healthy individuals and those with chronic diseases, with a majority being female. They achieved these results through the application of combined exercises (aerobic + resistance) with moderate intensity and a weekly frequency of two to three sessions, on average, for 12 weeks.

Simultaneously, the study by Brauer et al. [46], which included 9 studies with 440 participants aged 18 to 75 years old, examined aerobic, resistance, and combined exercise interventions in both acute and chronic sessions. The participants included sedentary individuals, athletes, and non-athletes, with a majority of the identified sample being female. The study highlighted a reduction in inflammatory levels, specifically decreased levels of IL-6, TNF-α, and senescent and memory effector T cells expressing CD45RA subsets (TEMRA). Notably, this reduction was observed only in the case of aerobic exercises when compared to resistance and combined exercises.

Haaland et al. [61], which included 17 studies with 794 participants aged 64.6 to 90.6 years old, also reported that aerobic exercises, performed by a sample primarily consisting of older females under healthy conditions, resulted in a decrease in IL-1β, IL-2, IL-6, IL-18, and TNF-α. However, the same effects were not observed with the practice of resistance and/or combined exercises. On the other hand, Khosravi et al. [50], which included 27 studies with 1190 participants aged 27 to 70 years old, mentioned that aerobic, resistance, and combined exercises, predominantly performed by women diagnosed with cancer, led to an increase in monocyte chemoattractant protein 1 (MCP-1) levels and a modest decrease in pro-inflammatory markers CRP and TNF. However, no significant changes were observed in serum levels of IL-6, IL-8, IL-1β, and INF-γ.

Considering the outcomes of resistance, aerobic, and combined (aerobic + resistance) exercises on IL-6 as a marker of immunosenescence, most systematic reviews indicate a statistically significant effect on reducing pro-inflammatory markers such as CRP, IL-6, and TNF-α with regular practice of aerobic physical exercises. It is important to note that the number of systematic reviews focusing on resistance exercises, particularly those with randomized and non-randomized clinical trials, was significantly smaller compared to aerobic exercises and combined exercises. This highlights the urgent need for conducting high-quality clinical trials specifically addressing the effects of resistance exercise in the context of immunosenescence and IL-6.

### 3.4. Evaluation of the Methodological Quality of Systematic Reviews

The assessment and grading of the methodological quality of the systematic reviews using the AMSTAR-2 checklist can be found in Table 4. Of the total 13 systematic reviews analyzed, 8 were rated as critically low quality [46,47,50,54,58,59,60,61]. Five systematic reviews were rated as low quality [43,44,45,50,52]. There were no systematic reviews considered to have moderate or high methodological quality.

## 4. Discussion

The purpose of this overview was to analyze the methodological quality of published systematic reviews that included randomized and non-randomized clinical trials in order to synthesize the evidence on IL-6 and immunosenescence in individuals who engage in aerobic and/or resistance exercise. For this purpose, a total of 13 systematic reviews were analyzed using the AMSTAR-2 tool, which is designed to assess the methodological quality of systematic reviews. All 16 items of the checklist were applied to each systematic review, considering both critical and non-critical items [69] to accurately classify their quality.

Out of the eight systematic reviews that were rated with critically low methodological quality, five of them focused on studies involving aerobic exercise [51,54,58,59,60] and three on combined exercise [46,47,61]. Of five systematic reviews classified as low methodological quality, two were of resistance exercise [44,45], one of aerobic exercise [52], and two of combined exercise [43,50], for a total of 13 systematic reviews.

Nine systematic reviews [44,45,47,50,51,52,54,58,60] incorporated the components of the PICO framework in their search questions and inclusion criteria (I.1). Only one study, Salimans et al. [45], provided a description of the method employed in the systematic review and possible justifications for any deviations, which was recorded in a protocol. This particular study was also registered in PROSPERO and had the appropriate information confirmed (I.2). All authors of the 13 systematic reviews analyzed mentioned the inclusion criteria for study designs in their texts (I.3). While most authors employed a comprehensive literature search strategy, none of the 13 studies met all the required criteria successfully (I.4).

Seven reviews explicitly mentioned that the selection of studies was conducted by at least two authors (I.5) [43,44,45,47,50,52,59]. However, four investigations did not provide information on the extraction of duplicate data (I.6) [46,51,54,59]. Regarding the listing and justification of excluded studies (I.7), this information was partially provided in three reviews [46,54,59]. Adequate details about the included studies (I.8) were reported partially in four studies [50,51,54,61], while one study did not provide this information [59].

Regarding the use of a satisfactory technique to assess the risk of bias in individual studies included in the review (I.9), all 13 reviews met this requirement. However, none of the 13 studies mentioned information about the funding sources for the included studies (I.10) [43,44,45,46,47,50,51,52]. In the systematic reviews that conducted a meta-analysis, the use of appropriate methods for statistical combination of results (I.11) was observed in only five studies [43,44,50,52,58]. The assessment of the potential impact of the risk of bias in individual studies on the results of meta-analysis or other evidence synthesis (I.12) was described in seven of the systematic reviews [43,44,45,50,52,58,60]. The risk of bias was not considered in individual studies when interpreting/discussing the review results (I.13) in four of the studies [47,51,54,61]. The provision of a satisfactory explanation and discussion of heterogeneity in the review results (I.14) was not found in three of the studies [47,50,61].

In the reviews that conducted quantitative synthesis, adequate investigation of publication bias and discussion of the likely impact on results (I.15) were not mentioned in six studies [46,47,50,54,59,61]. Mention of potential sources of conflict of interest and possible funding for conducting the reviews (I.16) was not reported in four systematic reviews [58,59,60,61]. Given the above, it was possible to identify several limitations in the analyzed systematic reviews. These limitations include the absence of protocol registration, insufficient information about the samples, heterogeneity among the included studies, and gaps in the analysis of publication bias, among others. These limitations collectively suggest a poor methodological quality of the studies.

However, it is important to highlight the advancements made in recent years regarding instruments that assess the methodological quality of systematic reviews. For instance, the AMSTAR tool was initially developed in 2007 and later updated in 2017 as AMSTAR-2 [69] by the same group of authors, introducing a more comprehensive and rigorous framework. Nevertheless, it is still common to come across recent studies that continue to utilize the original AMSTAR [70] or other variations, such as AMSTAR-R [71], published in 2010. This practice directly impacts the methodological evaluation and classification of systematic reviews since earlier instruments allow for flexibility in the evaluation criteria, potentially leading to divergent results based on the tool used. Therefore, researchers should be mindful of the potential factors that can influence the reliability of their conclusions [72,73].

To the best of our knowledge, this overview is unique in its comprehensive analysis of existing systematic reviews concerning the relationship between aerobic exercise, IL-6, and immunosenescence. This study stands out in its synthesis of the complex interactions and impacts of exercise on aging and inflammation, filling a significant gap in the literature. By consolidating diverse studies into a cohesive understanding, it provides invaluable insights for future research and practical applications in health management for aging populations. The significance of this study lies not only in its novel approach but also in its potential to guide effective strategies to mitigate the effects of aging and promote healthier, longer lives.

The studies analyzed revealed differences in the effects of exercise on IL-6 levels across different populations and contexts. In resistance exercises, Kim and Yeun [44] demonstrated a significant reduction in IL-6 levels in older adults with chronic conditions, while Salimans et al. [45] did not observe significant changes in healthy individuals. In aerobic exercises, Zheng et al. [52] observed a reduction in IL-6 levels in healthy adults, while Barros et al. [54] found a decrease in IL-6 levels in runners, suggesting aerobic exercise as a mediator in inflammatory processes. Additionally, Tong et al. [58] and Djalilova et al. [51] found variable effects of aerobic exercise in cancer patients. Regarding combined exercises, Yang et al. [43] observed reductions in IL-6, IL-10, CRP, and TNF-α levels, while Bautmans et al. [47] and Brauer et al. [46] found positive results in reducing IL-6 levels in healthy and chronically ill populations. Finally, Haaland et al. [61] and Khosravi et al. [50] highlighted the effects of aerobic exercise on elderly individuals and women with cancer, respectively. These findings underscore the importance of more high-quality clinical trials to better understand the effects of resistance exercise on immunosenescence and IL-6 levels.

Based on the results of the systematic reviews included in your study, it is evident that aerobic exercise significantly reduces inflammatory markers like IL-6, CRP, and TNF-α, suggesting its potential as an anti-inflammatory intervention [74]. However, the methodological quality of most reviews analyzed was rated as low or critically low. These findings emphasize the need for high-quality research to provide robust evidence on the effects of exercise on immunosenescence. This underscores the potential of exercise for managing inflammaging and highlights gaps in current research.

### 4.1. Strengths and Limitations

In terms of strengths, this study can be recognized as a pioneering effort in systematically gathering, evaluating, and synthesizing scientific evidence concerning the interplay between physical exercise, IL-6, and immunosenescence. The findings from current systematic review studies indicate that aerobic exercise holds promise as an anti-inflammatory intervention, effectively modulating the levels of IL-6, CRP, and TNF-α. However, the notable limitation lies in the methodological quality of the systematic reviews analyzed, underscoring the pressing need for robust and high-quality research in this field.

As for limitations, it is important to acknowledge that this study is subject to certain constraints. Firstly, the inclusion criteria were limited to studies published solely in English, potentially omitting relevant non-English literature. Furthermore, the scope was restricted to scientific articles, excluding other valuable document types. Additionally, the utilization of a contemporary methodological quality assessment tool for studies published prior to its development poses a limitation that should be taken into account when interpreting the results.

This study highlights the anti-inflammatory effects of aerobic exercise on aging-related biomarkers like IL-6, CRP, and TNF-α. It uncovers a need for higher-quality research in the field, emphasizing the importance of such studies for understanding inflammaging and immunosenescence. This research directs future efforts toward more detailed and methodologically sound studies, crucial for advancing knowledge in gerontology and developing effective interventions for aging populations.

### 4.2. Future Directions

Future investigations involving reviews of reviews should consider several perspectives to enhance the quality and breadth of the research. Firstly, comprehensive literature searches should be conducted, expanding beyond English-language scientific articles to include a wider range of databases and sources. Additionally, the inclusion of other types of documents, such as gray literature, would provide a more complete overview of the evidence. Researchers also should focus on specific populations and interventions, exploring the effects of physical exercise on immunosenescence in different age groups, individuals with specific chronic conditions, and athletes. Considering these perspectives, researchers can advance their understanding of the topic, inform evidence-based practices, and contribute to the development of tailored exercise recommendations.

The practical applications of this study have important implications for healthcare practices, especially in the management of aging populations. The demonstrated anti-inflammatory effects of aerobic exercise on markers like IL-6, CRP, and TNF-α offer a scientific foundation for creating specific exercise regimens for older adults. These results can guide public health policies, underscoring the importance of physical activity in mitigating inflammaging and enhancing overall health. The adoption of these findings could lead to improved strategies in the prevention and management of age-related diseases, underscoring the essential role of regular physical activity in promoting healthy aging.

## 5. Conclusions

Based on the studies examined, aerobic exercise emerges as a potential anti-inflammatory factor with the ability to positively influence the levels of IL-6, CRP, and TNF-α, particularly in the context of immunosenescence. However, it is crucial to recognize that the limited methodological quality of the systematic reviews analyzed highlights the pressing need for rigorous and high-quality studies. Such studies are essential to enhance information accessibility and facilitate evidence-based decision-making in healthcare.

## Figures and Tables

**Table 1:** The main information for constructing the PICO statement.

Acronym	Definition	Information
P	Population	Adolescents, adults, and elderly
I	Intervention	Exercise (including duration, modality, and intensity)
C	Comparison	No exercise
O	Outcomes	Positive effect on IL-6 as a biomarker of immunosenescence.

**Table 2:** Summary of the descriptors used in the search strategy.

Exercise
… OR Exercises OR Physical Activity OR Activities, Physical OR Activity, Physical OR Physical Activities OR Exercise, Physical OR Exercises, Physical OR Physical Exercise OR Physical Exercises OR Acute Exercise OR Acute Exercises OR Exercise, Acute OR Exercises, Acute OR Exercise, Isometric OR Exercises, Isometric OR Isometric Exercises OR Isometric Exercise OR Exercise, Aerobic OR Aerobic Exercise OR Aerobic Exercises OR Exercises, Aerobic OR Exercise Training OR Exercise Trainings OR Training, Exercise OR Trainings, Exercise AND…
Cellular Senescence
… OR Senescence, Cellular OR Cell Senescence OR Senescence, Cell OR Cell Aging OR Cellular Ageing OR Ageing, Cellular OR Aging, Cell OR Senescence, Replicative OR Cellular Aging OR Aging, Cellular OR Replicative Senescence OR Cell Ageing OR Ageing, Cell OR Aging OR Senescence OR Biological Aging OR Aging, Biological OR Immunocompetence OR Immunological Competence OR Competence, Immunological OR Competence, Immunologic OR Immunologic Competence AND…
Cytokines
… OR Cytokine OR Interleukins OR Interleukin OR Interleukin-6 OR Interleukin-6 receptors OR Interleukin 6 OR IL6 AND…
Inflammation
… OR Inflammations OR Innate Inflammatory Response OR Inflammatory Response, Innate OR Innate Inflammatory Responses
Systematic Review
…OR Systematic literature review OR Systematic critical review OR Systematic mixed studies review OR Systematic search and review OR Systematic meta-review OR Metanalysis OR Meta-analysis…
Aging
… OR Senescence OR Biological Aging OR Aging, Biological OR Aged OR Elderly…

**Table 3:** Characteristics of the studies included in the overview.

Author (Year) Reference	Total and Range of Study Participants/Settings	Number of Studies	Exercise	Meta-Analysis	Quality Assessment	Outcomes
Yang et al. (2023) [43]	A total of 853 (type 2 diabetics) (BMI ≤ 42, duration of disease ≥ 1 year); intervention: 44 years; control: 45 years; both sexes (balanced sample); age: 40 to 55 years >55 years	18 RCTs	C Aerobic, resisted, CON, HIIT; 3 weeks to 12 months	Y	Cochrane Collaboration (ROB)	↓ IL-6, IL-10, CRP, TNF-α
Kim; Yeun, (2022) [44]	A total of 539 (268—intervention group, 271 control: usual care/inactivity) 10 healthy elderly interventions and 8 diseases (metabolic syndrome, type 2 diabetes, cognitive impairment)	18 RCTs	R Intervention machines, elastic band; 6 to 32 weeks	Y	Cochrane Collaboration (ROB)	↓ IL-6, CRP, TNF-α, ↑ IL-10
Salimans et al. (2022) [45]	A total of 835 (284 adults); resistance exercise, 17 and 40 years; 8 resistance exercise, 60 and 85 years; healthy	30 (11 RCTs/6 NRCT/2 randomized control I/2 compared two I/exercise groups to each other in a non-randomized/8 single-intervention group with no control I/1 post-I only)	R Equipment: gymnastics and free weights; 3 to 12 weeks	N	Cochrane Collaboration (ROB2)	↑ NF-κB, MCP-1; * TNF-α, IL-6, IL-6R
Brauer et al. (2021) [46]	A total of 440. Acute exercise (3): running test (1), stationary bicycle (1), resisted (1); trained: rowing football, running, or resistance exercise. Sedentary: last 6 months, no more than 2 to 3x/or no more 150 min/week; 173 F; 66 M; 201 NI; 18 to 75 years.	9 (3 RCTs 4 RCTs, non-randomized or uncontrolled, 2 cohort studies)	C Acute exercise: running test, exercise bike; chronic sessions; 6 weeks to 6 months	N	Cochrane Collaboration (ROB2/ ROBINS-I)	↓ L-6, TNF-α, inflammation, cytokines
Bautmans et al. (2021) [47]	A total of 985 studies (8 with healthy patients, 1 in frailty and 4 with elderly diseases: cancer, on hemodialysis). 8 aerobic exercise (5 elderly with specific conditions/diseases); 7 resistance exercises (3 in healthy, 1 fragile, and 3 elderly with specific conditions/diseases). 1 Tai Chi in older adults/mild cognitive impairment; 70 F, 249 M, 666 NI (estimated); 56 to 83 years.	19 RCTs/3 NRCT/1COR (19 randomized controlled trials; crossover; 3 non-randomized controlled intervention studies; 1 randomized intervention)	C Resistance exercise, aerobics, and Tai Chi; 3 weeks to 12 months	N	NICE	↓ IL-6, CRP, TNF-α
Khosravi et al. (2019) [50]	A total of 1190 cancer survivors (I: exercise—age 54.37; control—age 56.71) (13 combined exercise; 5 aerobic; 3 resisted). 5 yoga (n = 2), Tai Chi (n = 3), control habitual care (n = 17), educational I (n = 3), therapy (n = 2), health assessment (n = 1), counseling (n = 1), relaxation (n = 1), and oral I (n = 1); 857 F, 40 M, 93 NI (estimated); 27 to 70 years.	27 RCTs	C Combined aerobic exercise, resisted, yoga, Tai Chi; 2 to 104 weeks	Y	PeDro	↓ M.P.I, CRP, TNF; ↑ MCP-1; * IL-6, IL-8, IL-1β, INF-γ
Djalilova et al. (2019) [51]	A total of 937 cases of breast cancer, colorectal, heart failure, and hypertension (5 studies with controls on the waiting list and 4 usual or untreated care, 2 education, 1 time and attention, 2 three-arm intervention comparing yoga with 2 control groups); 706 F, 231 M; 35 to 66 years, average 48.54.	15 (10 randomized controlled trials; RCT/3 quasi-experimental, 2 single-group pre-posts)	A Types of yoga: Hatha yoga; 2 to 8 weeks	N	Quality Rating Scale	↓ Inflammation in several chronic conditions
Zheng et al. (2019) [52]	A total of 1250 healthy aerobic exercises (Tai Chi intervention groups (1), treadmill (1), step on the bench (1), multicomponents (5): 1062 F, 188 M, 3 NI (estimated); 40 to 95 years.	11 RCTs	A Tai Chi, treadmill, step on the bench, multicomponent; 2 to 12 months	Y	Cochrane Collaboration	↓ IL-6, CRP, TNF-α
Barros et al. (2017) [54]	A total of 1421 marathoners, recreational runners, sedentary, beginners, and only healthy participants: 163 F, 1234 M, 24 NI; average age: 39.16.	51 RCT/NRCT	A Marathon, ultramarathon, half marathon, distance protocols (42.195, 21.1, 12, 10 km and treadmill, chronic exercise; 24 h to 6 days	N	Downs and Black scale (Modified)	↓ IL-6, TNF-α, ↑ IL-10
Tong et al. (2013) [58]	A total of 276 (only for interventions with physical exercises); cancer/survivors walking, Tai Chi, Qigong, and aerobic exercise (moderate and low intensity); both sexes; 41.5 to 70.43 years.	76 RCTs (11 physical exercises, 5 multiple interventions); 54 psychological/cognitive therapies, 5 diets, 6 multiple strategies combining 2/3 of the interventions.	A walking, Tai Chi, Qigong, aerobic exercise (moderate and low intensity); 4 days to 6 months	Y	Instrument not informed	→ CD4+, CD4+/CD8+, CD3+, NKCA, IL-2; * E.C IL-6, CD8+, TNF-α
Ploeger, 2009 [59]	A total of 316. Asthma, diabetes mellitus 1 and 2, multiple sclerosis, chronic obstructive pulmonary disease, and healthy control. I (chronic exercise in adults and acute in children); control with 9–110 chronic disease (mean: 34.19); placebo therapy: 12/26; No I: 14/26. (estimated); sex: NI; ≤18 and >19 years old.	19 RCTs (12 studies in adults and 7 studies in children * (* deleted from this overview)	A Acute and chronic cycling; 8 to 12 weeks	N	PeDRO	↑ IL-6, TNF-α in chronic diseases, ↓ after 2 h of acute exercise/↓ IL-6, TNF-α healthy control
Ng; Tsang, (2009) [60]	A total of 796 (groups 9–115) have chronic diseases: hypertension, fibromyalgia, cancer, and neurological problems. (average: 35.06 people/group/Qigon), (estimated); sex: NI; 18.5 to 77.5 years.	26 RCTs	A Qigong and variations, walking; 3 to 12 weeks	Y	Jadad	↓ IL-6, ↑ white blood cells, lymphocytes
Haaland et al. (2008) [61]	A total of 794 (estimated total) (the sample size ranged from 13 to 190) were healthy. Water exercises, calisthenics, flexibility/toning, strength (upper and lower), 572 F, 201 M, 31 NR (estimated), 64.6 to 90.6 years.	17 RCTs (17 studies and 16 cohorts of participants)	C water exercises, calisthenics, flexibility/toning, strength (top and bottom); 8 weeks to 43 months	N	Jadad	↓ IL-6, IL-1b, IL-2, IL-18, TNF-α

Notes: RCT: Randomized Clinical Trial; NRCT: Non-Randomized Clinical Trial; COR: Randomized Crossover; I: Intervention; Y: Yes; N: No; R: Resistance; A: Aerobic; C: Combined (aerobic + resistance): CON: Continuous; HIIT: High-Intensity Interval Training; CRP: C-Reactive Protein; IL: Interleukin; IL-1β: Interleukin 1 beta; TNF: Tumor Necrosis Factor; TNF-α: Tumor Necrosis Factor Alpha; M.P.I: Pro-Inflammatory Markers; E.C: Combined Effects; NF-κB: Nuclear Factor kappa B; IL-6R: Interleukin 6 Receptor; MCP-1: Monocyte Chemoattractant Protein 1; INF-γ: Interferon-gamma; NKCA: Natural Miller Cell Activity: * No significant change; → Significant effect: ↑ increase; ↓ decrease.

**Table 4:** Quality assessment of systematic reviews by AMSTAR-2.

	AMSTAR-2																
Author/Year	I.1	I.2	I.3	I.4	I.5	I.6	I.7	I.8	I.9	I.10	I.11	I.12	I.13	I.14	I.15	I.16	GC
Yang et al., 2023 [43]	N	N	Y	P	Y	Y	P	Y	Y	N	Y	Y	Y	Y	Y	Y	L
Kim; Yeun, 2022 [44]	Y	N	Y	P	Y	Y	Y	Y	Y	N	Y	Y	Y	Y	Y	Y	L
Salimans et al., 2022 [45]	Y	Y	Y	P	Y	Y	Y	Y	Y	N	N	Y	Y	Y	Y	Y	L
Brauer et al., 2021 [46]	N	N	Y	P	N	N	Y	Y	Y	N	N	N	Y	Y	N	Y	CL
Bautmans et al., 2021 [47]	Y	N	Y	P	Y	Y	Y	Y	Y	N	N	N	N	N	N	Y	CL
Khosravi et al., 2019 [50]	Y	N	Y	P	Y	Y	Y	P	Y	N	Y	Y	Y	Y	Y	Y	L
Djalilova et al., 2019 [51]	Y	N	Y	P	N	N	Y	P	Y	N	N	N	N	N	N	Y	CL
Zheng et al., 2019 [52]	Y	N	Y	P	Y	Y	Y	Y	Y	N	Y	Y	Y	Y	Y	Y	L
Barros et al., 2017 [54]	Y	N	Y	P	N	N	Y	P	Y	N	N	N	N	Y	N	Y	CL
Tong et al., 2013 [58]	Y	N	P	N	Y	P	Y	Y	N	Y	Y	Y	Y	Y	Y	N	CL
Ploeger, 2009 [59]	N	N	Y	P	Y	N	Y	N	Y	N	N	N	Y	Y	N	N	CL
Ng; Tsang, 2009 [60] Haaland et al., 2008 [61]	Y Y	N N	Y Y	P P	N N	Y Y	Y P	Y P	Y Y	N N	N N	Y N	Y N	Y N	Y N	N N	CL CL

Notes: I: Item; GC: Overall Rating; Y: Yes; N: No; P: Partially Yes; L: Low; CL: Critically Low.

## Data Availability

Data are contained within the article.
